# Perfluorosulfonic Acid Membranes with Short and Long Side Chains and Their Use in Sensors for the Determination of Markers of Viral Diseases in Saliva

**DOI:** 10.3390/membranes13080701

**Published:** 2023-07-27

**Authors:** Anna V. Parshina, Ekaterina Yu. Safronova, Svetlana A. Novikova, Nastasia Stretton, Anastasia S. Yelnikova, Timur R. Zhuchkov, Olga V. Bobreshova, Andrey B. Yaroslavtsev

**Affiliations:** 1 Department of Analytical Chemistry, Voronezh State University, 394018 Voronezh, Russia; parshina_ann@mail.ru (A.V.P.); anastasia_elnikova@outlook.com (A.S.Y.); timurzuckov65617@gmail.com (T.R.Z.); bobreshova@chem.vsu.ru (O.V.B.); 2 Kurnakov Institute of General and Inorganic Chemistry, Russian Academy of Sciences, 119991 Moscow, Russia; safronova@igic.ras.ru (E.Y.S.); svetlana_novi@mail.ru (S.A.N.); stretton.nastasia@gmail.com (N.S.)

**Keywords:** PFSA membrane, Nafion, Aquivion, ion transport, Donnan potential, potentiometric multisensory system, saliva, low-molecular-weight marker, viral disease

## Abstract

The development of accessible express methods to determine markers of viral diseases in saliva is currently an actual problem. Novel cross-sensitive sensors based on Donnan potential with bio-comparable perfluorosulfonic acid membranes for the determination of salivary viral markers (*N*-acetyl-*L*-methionine, *L*-carnitine, and *L*-lysine) were proposed. Membranes were formed by casting from dispersions of Nafion or Aquivion in *N*-methyl-2-pyrollidone or in a mixture of isopropyl alcohol and water. The influence of the polymer equivalent weight and the nature of dispersing liquid on water uptake, ion conductivity, and slope of Donnan potential for the membranes in H^+^ and Na^+^ form was investigated. The varying of the sorption and transport properties of perfluorosulfonic acid membranes provided a change in the distribution of the sensor sensitivity to *N*-acetyl-*L*-methionine, *L*-carnitine, and *L*-lysine ions, which was necessary for multisensory system development. The simultaneous determination of three analytes, and the group analysis of them in artificial saliva solutions, was performed. The errors of *N*-acetyl-*L*-methionine and *L*-carnitine determination were 4–12 and 3–11%, respectively. The determination of *L*-lysine was complicated by its interaction with Ca^2+^ ions. The error of the group analysis was no greater than 9%. The reverse character of the viral markers’ sorption by the membranes provided long-term sensor operation.

## 1. Introduction

Technological processes based on the use of membrane technologies fully meet the requirements of sustainable development and green chemistry, which makes them more in demand in the modern world. Membrane technologies are widely used in water treatment, water purification, gas separation, treatment of industrial effluents and products, food and pharmaceutical industries, and alternative applications [1,2,3,4,5,6,7,8,9]. One of the most important classes of membrane materials is polymer membranes, because, in addition to their transport properties and selectivity, their important functional characteristics are strength, elasticity, and stability [10]. Perfluorinated materials have the best chemical stability among polymeric membranes, so they are constantly at the center of the attention of researchers and engineers working in the field of membrane technology [11,12,13,14].

Ion-exchange membranes based on perfluorosulfonic acid (PFSA) polymers are used in energy generation and storage systems, in particular in fuel cells, electrolyzers, redox flow batteries, and metal-ion batteries [7,15,16,17,18,19,20]. PFSA membranes such as Nafion^®^ with relatively long side chains (LSC) formed by ether and fluorocarbon groups terminating in functional sulfogroups (-O-CF_2_-CF(CF_3_)-(CF_2_)_2_-SO_3_H) are preferably used for this purpose. Such materials have high proton conductivity along with high selectivity [21]. The functional properties of Nafion membranes do not fully meet the requirements for practical applications, which leads to an interest in finding alternative materials or approaches to optimize the properties of existing materials [7]. One solution to increase the proton conductivity and the operating temperature of such materials is to switch to PFSA membranes with a short side chain (SSC), in particular Aquivion [22]. Its side chain includes two carbon atoms (-O-CF_2_-CF_2_-SO_3_H). This change leads to the possibility of obtaining stable films from polymers with a high content of functional sulfogroups and a low equivalent weight (EW) (average polymer mass per mol of functional group) due to a higher degree of crystallinity of the polymer [23,24].

The key issue in creating membranes based on PFSA polymers with high conductivity and selectivity is to control the microstructure and form a branched system of pores and channels with good interconnectivity [21,25,26,27,28,29,30,31]. The difference in the nature of the backbone and side chains of the polymer leads to the formation of clusters of hydrophilic sulfogroups. As a result of their hydration, a system of pores connected by channels is formed in the membrane [32,33]. The size of the pores and channels as well as the interconnectivity of the system determines the conductivity of the materials. The formation of membranes by casting from polymer dispersions makes it possible to control their microstructure by changing the conditions of film formation (dispersion composition, temperature, and duration of dispersant removal) and to vary the mechanical properties, water uptake, proton conductivity, and selectivity of cation transport [34,35,36,37]. It was shown that membranes based on SSC and LSC PFSA polymers with an optimized microstructure have advantages when used as an electrolyte in a hydrogen–air fuel cell due to higher proton conductivity [28]. The most suitable dispersing liquids for PFSA polymers are aprotic polar solvents, as well as mixtures based on water and low-molecular-weight alcohols [36,38].

PFSA membranes have found alternative applications in recent decades, in particular, in analytical chemistry. Nafion membranes are used as the hydrophilic component of optical sensors for the determination of humidity [39,40,41,42]. This approach is based on the change in the thickness and refractive index of Nafion film due to a dependent relationship with the environmental humidity (in this case, Nafion is the sensitive layer) [39,40,41] or with the protonation–deprotonation reaction of an optically active substance that is introduced to the Nafion pores [42]. In the case of electrochemical sensors, Nafion can be used for the stabilization of nanoparticles of the catalytic layer, protection of the sensor material from fouling, as well as for the immobilization of aptamers (it is relevant to electrochemical biosensors). At the same time, the sensor specificity is provided by the properties of the catalytic layer or biolayer, so the analyte nature can be different. Materials based on a Nafion membrane and Pt/C were proposed for the amperometric determination of hydrogen [43]. Voltammetric sensors based on Nafion/TiO_2_ composites with a low limit of detection (LOD) of pesticides (phenyltrothion) are known [44]. A glass carbon electrode was modified by a Nafion film containing multi-walled carbon nanotubes (MCNTs) for the voltammetric determination of histamine [45]. Composites based on Nafion and MCNTs in an aptasensor with a voltammetric type of detection were developed for the ochratoxins’ determination [46]. A solid-contact potentiometric sensor, consisting of an Al_2_O_3_ substrate covered with RuO_2_, a thin layer of Ta_2_O_5_, and Nafion film, was suggested for the pH control in beverages [47]. One of the fields of the use of optical and electrochemical sensors based on PFSA polymers is medical diagnostics. In this case, the determined components of physiological media are generally low-molecular-weight markers of various diseases or toxic and illicit drugs. For example, Nafion was a sensor component for the determination of nitric oxide, ammonia, uric acid, *L*-tyrosine, choline in blood [48,49,50,51,52,53], uric acid, *L*-tyrosine, sarcosine, creatinine in urine [51,52,54,55], phenylalanine, creatinine in saliva [56,57], sodium ions, and total ion content in sweat [58,59], as well as glucose in blood [60,61], saliva [62], interstitial fluid [63], and sweat and tears [64,65] as disease markers. Sensors based on composite materials with Nafion were developed for the determination of *L*-tryptophan [66], salbutamol [67], metformin [68], clozapine [69], itopride [70], and clioquinol [71] in physiological media. The high biocompatibility of PFSA polymers, in addition to the previously mentioned properties, is important for biomedical applications. For instance, it allows for their usage as a matrix for wearable and implanting sensors for in vivo analysis [48,56,58,59,60,63,64].

A possibility for the use of PFSA membranes as a sensing material of potentiometric sensors, whose analytical signal is the Donnan potential (DP), has been shown for the determination of amino acids [72,73]. Amino acids and their derivatives are relevant analytes due to their antiviral, and general restorative properties, as well as their overproduction in the human organism in response to viral and cancer diseases. DP-sensors have no specific selectivity for specific analytes, but can be used to organize multisensory systems with cross-sensitivity [72,73,74,75,76,77,78]. The DP-sensor sensitivity to different components of a studied medium depends on the sorption ability of PFSA membranes to them. The use of approaches based on the modification of the PFSA membrane interpore space and the introduction of nanoparticles of various dopants into them provided the possibility of directional changes in the DP-sensor sensitivity, depending on the nature of organic ions. Good results were obtained for the analysis of pharmaceutical media when PFSA membranes modified by oxides with a functionalized surface [73], functionalized MCNTs [74], or conducting polymers [75] were used in DP-sensors. At the same time, different preliminary treatments of PFSA membranes were effective for the varying of DP-sensor sensitivity as well [72,73,75]. It is of great scientific and practical interest to search for the possibilities of the simplification of the composition and preparation techniques of functional materials. Therefore, in this work, a change in the number of functional groups and membrane microstructure due to the use of various PFSA polymers and casting of membranes from different dispersing liquids was investigated as the approaches to the change in the DP-sensor sensitivity to salivary markers of viral diseases.

Thus, the goal of this work is to study membranes based on PFSA polymers with SSC and LSC (commercial and obtained by casting from dispersions of different compositions) as materials of cross-sensitive DP-sensors for determining markers (*N*-acetyl-*L*-methionine, *L*-carnitine, and *L*-lysine) of viral diseases in saliva.

## 2. Materials and Methods

### 2.1. Materials and Reagents

The following reagents were used: *N*-methyl-2-pyrollidone (NMP) (Merck, Darmstadt, Germany), isopropyl alcohol (IPA) (special purity, Chimmed, Moscow, Russia), lithium hydroxide (≥99.0%, Sigma Aldrich, St. Louis, MO, USA), hydrochloric acid (special purity, 35–38%, Chimmed, Moscow, Russia), nitric acid (special purity, 60%, Chimmed, Moscow, Russia), hydrogen peroxide special (purity, 30%, Chimmed, Moscow, Russia), potassium chloride (reagent grade, Chimmed, Moscow, Russia), potassium hydroxide (reagent grade, Chimmed, Moscow, Russia), sodium chloride (reagent grade, Chimmed, Moscow, Russia), calcium chloride (reagent grade, Chimmed, Moscow, Russia), sodium bicarbonate (reagent grade, Chimmed, Moscow, Russia), *N*-acetyl-*L*-methionine (AM) (99%, J&K Scientific, San Jose, CA, USA), *L*-carnitine (CT) (>98%, Alfa Aesar, Haverhill, MA, USA), *L*-lysine (LS) (>99%, Alfa Aesar, Haverhill, MA, USA), deionized water (resistance 18.2 MOhm).

The membranes used were Nafion^®^ 212 (The Chemours Company, Wilmington, DE, USA) and Aquivion E87-05S (Solway, Brussels, Belgium). Nafion^®^ 212 film (The Chemours Company, Wilmington, DE, USA) and Aquivion PW79S powder (Solway, Brussels, Belgium) were used to obtain PFSA polymer dispersions. The main characteristics of the materials used are shown in Table 1.

### 2.2. Conditioning and Pretreatment of Membranes

Before the experiments, the membranes were conditioned according to the procedure described in [76]. The membranes were boiled in a 3% hydrogen peroxide solution for 1 h, then successively immersed in a 7 M nitric acid solution at room temperature for 30 min, in a 1 M hydrochloric acid solution at 80 °C for 1 h, and in water at 90 °C for 1 h. The transfer into Li^+^ and Na^+^ forms was carried out by immersion in 1 M solution of the corresponding alkali with threefold replacement of the solution every 2 h. The obtained membranes were washed with deionized water and stored in it.

### 2.3. Formation of Polymer Dispersions

NMP and a mixture of IPA with water in a 4:1 ratio (IPA-H_2_O 80-20) were used as dispersing liquids.

The sample names and conditions for obtaining the dispersions are shown in Table 2. To obtain the dispersions, the PFSA polymers were pre-dried in an OV-11/12 vacuum oven (Jeio Tech, Daejeon, Korea) at 50 °C for 6 h. Dry samples were placed in a round-bottom flask together with the dispersing liquid at a ratio of either 2.5 or 5 wt% of polymer. Mixture was carried out with constant stirring at a certain temperature. The use of the Nafion 212 membrane in the Li^+^ form is due to its higher thermal stability compared to the H^+^ form. The conditions for obtaining the dispersions were chosen experimentally so that the polymer dispersed at the lowest possible temperature.

Dispersion of PFSA polymer with an SSC from Aquivion 87 film is more difficult compared to Nafion 212 film with an LSC. As a result, a dispersion with high viscosity (*η*_disp_. = 76.4 mPa·s for 2.5 wt% dispersion in NMP) is formed, indicating high PFSA polymer agglomeration. Therefore, dispersions obtained from Aquivion powder were used to obtain membranes with an SSC by a casting procedure.

### 2.4. Membrane Formation by Casting Procedure

To form the membranes by casting, the obtained dispersions were poured onto a glass surface and heated gradually to remove the dispersing liquid. In the case of NMP, the samples were successively heated in air at 60 °C for 3 h and in vacuum at 120 °C for 6 h. In the case of IPA-H_2_O, the samples were heated in air at 40 °C for 3 h and in a vacuum at 80 °C for 3 h. The weight of the residue after removal of the dispersing liquid was 4.9–5.0 wt% of the dispersion mass for samples with a polymer concentration of 5.0 wt% and 2.4–2.5 wt% of the dispersion mass for samples with a polymer concentration of 2.5 wt%. All films obtained after the removal of the dispersing liquid were subjected to hot pressing at 5 MPa at 110 °C for 3 min to provide better strength, then chemically treated to bring them to standard conditions and transfer them into H^+^ form. For this purpose, they were successively treated at room temperature first twice with 5 wt% HCl solution for 1.5 h, then washed with deionized water until the reaction for Cl^−^ ions disappeared.

Thus, six membrane samples were investigated in this work: commercial Nafion 212 and Aquivion 87 and laboratory cast Nafion-IPA-H_2_O, Nafion-NMP, Aquivion-IPA-H_2_O, and Aquivion-NMP.

### 2.5. Membrane Characterization

The density of dispersing liquids and dispersions was determined using a Densito portable density meter (Mettler Toledo, Greifensee, Switzerland) at 25 ± 0.1 °C. Viscosity was determined using a vibrating viscometer SV-1A (A&D, Tokyo, Japan) at 25 ± 0.2 °C. The value of dynamic viscosity (*η*, mPa∙s) was calculated from the ratio of the experimentally obtained viscosity to the liquid density. The viscometer was calibrated at two points using 5 and 10 mPa∙s viscosity standards (Brookfield, Toronto, ON, Canada). Viscosity was calculated from the arithmetic mean of three independent experiments.

The ion-exchange capacity of the membranes (IEC, mg-eq/g) was determined by direct acid–base titration using an Econix Expert 001 pH-meter (Econix-Expert, Moscow, Russia) to establish the equivalence point. Polymer powder or membrane in H^+^ form was preheated at 150 °C for 30 min to remove water. The sample weight of ~0.3 g was stored in 50 mL of 0.1 M NaCl solution for 6 h with constant stirring. After that, the salt solution with the membrane was titrated with 0.05 M NaOH solution. The IEC was calculated relative to the weight of the dry sample.

The water uptake of the membranes was determined gravimetrically. The membrane in the appropriate ionic form was dried at 50 °C in a vacuum oven for 24 h and weighed (m_dry_, g). The sample was placed in deionized water for 24 h and then weighed (m_hydr_, g). Water uptake (W, %) was calculated by the formula:

(1) $$W=\frac{m_{\mathrm{hydr}}-m_{\mathrm{dry}}}{m_{\mathrm{dry}}}\times 100$$

The number of water molecules per functional group (*λ*(H_2_O/−SO_3_^−^)) was calculated according to the formula:

(2) $$\lambda \left(H_{2}O/-{\mathrm{SO}}_{3}^{-}\right)=\frac{W}{M\left(H_{2}O\right)\times \mathrm{IEC}}\times 10,$$

where M(H_2_O)-molar mass of water, g/mol.

The ionic conductivity of the membranes was studied in contact with deionized water at 25 °C using an E-1500 AC bridge (Elins LLC, Chernogolovka, Russia) in the frequency range from 10 to 3 MHz on symmetrical carbon/membrane/carbon cells with an active surface area of 0.78 cm^2^. The value of conductivity (*σ*, mS/cm) was calculated from the resistance found from the impedance hodograph at the cutoff along the axis of active resistance.

FTIR spectra of membranes were registered using a Vertex 70 FTIR spectrometer (Bruker, Mannheim, Germany) attenuated total reflection mode. Before the measurements, the membranes in the swollen state were maintained under 95% relative humidity for 24 h.

To estimate the DP at the interface of the membranes in the H^+^ and Na^+^ forms with HCl and NaCl solutions, the cell, described in [73], was used. The electrochemical chain for the DP estimation on the membrane interface with the test solution was the following:

(3) Ag|AgCl, sat. KCl|1 M XCl|membrane|10^−5^–10^−1^ M XCl|sat. KCl, AgCl|Ag,

where X—H^+^ or Na^+^, 1M XCl—reference solution, and 10^−5^–10^−1^ M XCl—test solution.

One section was used for the test solution, while several sections were used for the reference solution. Each membrane connected the section with the test solution and one of the sections with the reference solution. The voltage was measured between a reference electrode, immersed in the test solution, and electrodes, immersed in the sections of the reference solution. ESr-10103 silver chloride electrodes (Econix-Expert, Moscow, Russia) were used. The measurements were performed using an analog-to-digital transmitter of multichannel potentiometer. The minimization of all interface differences in potential and diffusion potentials in the chain (3), apart from the DP at the membrane interface with the test solution, was achieved due to the following techniques. The distance between the test and reference solutions was significantly greater in comparison to the traditional cells for the measurement of the membrane potential. This distance corresponded to the membrane length and was ~6 cm. The depth of the immersion of the membrane ends into the solutions was no more than 0.3 cm. As a result, equilibrium in the reference solution/membrane/test solution system was not reached, while quasi-equilibrations at the membrane interfaces with the solutions were established. Time of quasi-equilibration in the system was no greater than 1 min. That was proved by experimental chronopotentiometric measurements. Under such conditions, transmembrane transfer can be omitted since the time of the electrolyte diffusion through a membrane with diffusion permeability of around 10^−7^ cm^2^/s exceeds the time of reaching the constant value of the measuring voltage by orders of magnitude. The diffusion potential in the surface layer of a membrane, contacting a reference solution with a high concentration, as well as the DP at this interface, was eliminated due to the closeness of the concentrations of the inner and outer solutions. The Donnan exclusion at the membrane interface with the dilute test solution was well maintained.

### 2.6. Preparation of Artificial Saliva Solution and Measurements with DP-Sensors

The analytes were AM and CT amino acid derivatives and LS amino acid since it is important to control their levels upon COVID-19 monitoring, according to [77]. Artificial saliva solutions were used as analysis objects because the determination of salivary viral markers can be used for the purposes of non-invasive diagnostics. Ringer’s solution (9.0 g/L NaCl, 0.4 g/L KCl, 0.1265 g/L CaCl_2_, 0.2 g/L NaHCO_3_) was used as an artificial saliva solution.

The cell, described in Section 2.5, was used for the estimation of the DP-sensor responses. Membranes were used in the Na^+^ form to decrease the influence of the supporting electrolyte, which was in the composition of the artificial saliva solutions, on the DP-sensor response. The reference solution was 1 M NaCl. ES-10301/4 glass electrode (Econix-Expert, Moscow, Russia) was additionally immersed into the section with the test solution. Its response was measured relative to the reference electrode, immersed into the same section. This was necessary to control the pH of the solution since it was taken into account upon calibration of DPsensors. Owing to that, an additional pH correction of the analysis object was not required.

**Figure 1:**
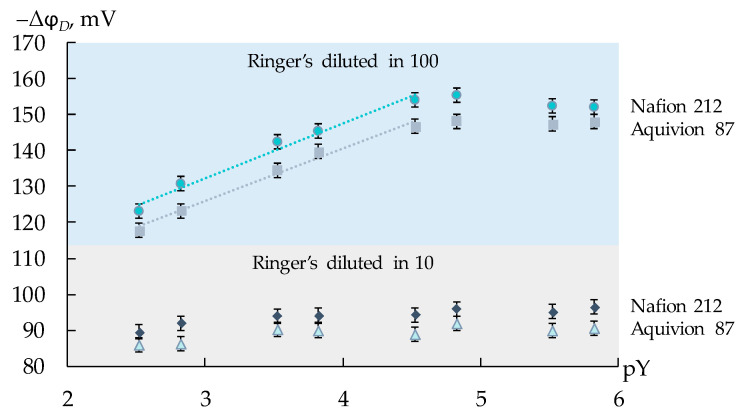
Dependence of the DP-sensor response on the negative decimal logarithm of the total concentration of AM^+/±^, CT^+/±^, and LS^+^ ions (pY) in artificial saliva solutions diluted 10 and 100 times.

The range of the working concentrations of the supporting electrolyte and the analytes was established through the experiment. Ringer’s solution was diluted 10 and 100 times, and weighed amounts of the analytes were added for the preparation of the equimolar test solutions with concentrations of each analyte ranging from 5 × 10^−6^ to 1.0 × 10^−3^ M. The dependence of the response of DP-sensors based on the commercial membranes on the negative decimal logarithm of the total concentration of the viral markers in the solution was established. Minimum dilution, characterized by the sensitivity of DP-sensors, which was enough for the quantitative analysis, was in 100 times (Figure 1). For these solutions, the concentration dependence of the DP-sensor response was linear in the analyte concentrations ranging from 1.0 × 10^−5^ to 1.0 × 10^−3^ M (Figure 1).

Thus, calibration characteristics of DP-sensors were established in the solutions with the constant concentration of the supporting electrolyte (100-fold dilution of the Ringer’s solution). The concentrations of three viral markers were varied in different ratios and ranged from 1.0 × 10^−5^ to 1.0 × 10^−3^ M. The pH values of the test solutions were 3.23–5.81. In this pH range, AM and CT were in the forms of cations and zwitter ions (furthermore, the equilibrium forms were designated as AM^+/±^, CT^+/±^), while LS was in the form of monovalent cations (LS^+^).

Multidimensional regression equations, which took into account changes in the concentration of each analyte upon their simultaneous presence (Equation (4)) and total concentration of the analytes (Equation (5)), were obtained. The pH influence on the analytical signal was taken into account in both equations. The adequacy of the regression equations and the significance of their coefficients were estimated using the standard algorithms.

(4) $${\Delta \varphi }_{D}=b_{0}+b_{1}\times \mathrm{pAM}+b_{2}\times \mathrm{pCT}+b_{3}\times \mathrm{pLS}+b_{4}\times \mathrm{pH},$$

(5) $${\Delta \varphi }_{D}=b_{0}+b_{1}\times \mathrm{pY}+b_{2}\times \mathrm{pH},$$

where ∆φ*_D_* (mV)—the DP-sensor analytical signal; pAM, pCT, pLS—the values of the negative decimal logarithm of the concentration AM^+/±^, CT^+/±^, and LS^+^ ions in the test solution; pY—the values of the negative decimal logarithm of the total concentration of AM^+/±^, CT^+/±^, and LS^+^ ions in the test solution; *b*_i_ (mV/p*c*)—the sensitivity coefficient to each ion or group of ions.

Calibration equations in the form of Equation (4) were used for the determination of AM^+/±^, CT^+/±^, and LS^+^ ions in the artificial saliva solutions upon their simultaneous presence. For this purpose, the responses of three DP-sensors and the pH were measured in the analysis object, and the system of three regression equations in the form of Equation (4) was solved. The equations in the form of Equation (5) were used for the group analysis of ions of the viral markers. In this case, the value of the pH and the response of one of the DP-sensors were enough. The predictive ability of a system of calibration equations is greater the higher the sensitivity coefficients (*b*_i_, mV/p*c*) and the lower the scatter (*ε*, %) between experimental and predicted values of the sensor analytical signal. The predictive ability of the system of calibration equations also depends on the correlation between the responses of single sensors. The errors of the analysis were estimated by the “added-found” method. The “3*σ*” rule was used for the estimation of LODs of the analytes in artificial saliva solutions.

### 2.7. Regeneration of Membranes

To prevent the membranes from fouling, they were washed with deionized water between each measurement. After a series of measurements (~50), the membranes were immersed in a 0.1 M NaCl solution for 1 h, then washed with deionized water and stored in it. After long-term use of the membranes, they were equilibrated with a 1 M NaCl solution for regeneration.

## 3. Results and Discussion

### 3.1. Membrane Characteristics

The viscosity of the dispersions of PFSA polymers depends on their EW, concentration, and nature of dispersing liquids. The viscosity of dispersions in NMP is lower than in IPA-H_2_O 80-20 (Table 2). This difference is due to peculiarities in the polymer-dispersing liquid and polymer–polymer interactions, as well as to the shape of macromolecules in the dispersion. According to the small-angle X-ray neutron scattering and atomistic molecular dynamics data, PFSA dispersions in aprotic solvents, such as NMP, have morphology, the closest to the real solution [78,79]. Due to the high affinity of the chains of PFSA polymer to the aprotic solvents, macromolecules are not aggregated and represent the conformation of chaotic random coils of a few nanometers in size. This dispersion is the closest to the true solution. With this morphology, the hydrodynamic resistance is low and the degree of agglomeration of macromolecules is low, which leads to a low viscosity of polymer dispersions. According to [78,79], macromolecules of PFSA polymers in contact with a water–alcohol mixture are self-organized as highly solvated large particles of about 200 nm in size with a high degree of agglomeration. Therefore, the viscosity of such dispersions is higher. The viscosity of Aquivion-based dispersions is higher than that of Nafion-based dispersions, especially in the water–alcohol mixture. One reason for this may be the higher degree of agglomeration of macromolecules in Aquivion-based dispersions due to the higher number of functional -SO_3_^−^-groups.

A decrease in the EW of PFSA polymer leads to an increase in the IEC of the membranes (Table 3) due to an increase in the number of functional groups. For membranes obtained by casting from Nafion dispersions, the IEC is virtually unchanged compared to the initial Nafion 212. The IEC of Aquivion membranes obtained by casting is slightly lower compared to that of Aquivion PW79S powder (1.26 mg-eq/g). The most likely reason for this is the reduced availability of functional sulfogroups in the material as a membrane. Also, the reason for obtaining an underestimated value of IEC may be a certain amount of water bound with sulfogroups, which remains even after drying [80].

The water uptake and ionic conductivity of membranes in H^+^ and Na^+^ ion form in contact with water were studied (Table 3 and Table 4). The water uptake of membranes in H^+^ form is higher than in Na^+^ form. The weight fraction of water in membranes with an SSC is higher than that of membranes with an LSC (Table 3). Since water sorption by PFSA membranes is determined by the number of functional groups, it is reasonable not to compare the weight fraction of water in the samples, but rather the amount of water per sulfogroup. Among commercial samples, the water uptake of the Aquivion 87 membrane is higher than that of Nafion 212. Hydration of PFSA membranes involves expansion of hydrophilic clusters. This process is limited by the hydrophobic matrix [16]. Increasing the number of side chains in the Aquivion 87 membrane compared to Nafion 212 leads to a decrease in the specific fraction of polymer chains and a decrease in the degree of crystallinity of the polymer. As a result, the membrane matrix’s ability to deform during swelling increases, and a larger amount of water is sorbed.

When casting membranes from PFSA polymer dispersions, the water uptake increases compared to a similar commercial sample (Table 3). For example, the water uptake of the Nafion-IPA-H_2_O and Nafion-NMP membranes is 1.5 times higher than that of Nafion 212. When casting membranes from polymer dispersions, the mobility of the polymer chains remains relatively high even after the removal of a solvent [35]. This allows for the sorption of large amounts of water. As a result, the water uptake of membranes cast from Nafion dispersions is even higher than that of the Aquivion 87 membrane, which has a higher IEC. The polymer morphology in a dispersion depends on the nature of the dispersing liquid. This affects the process of microstructure formation and results in a system of pores and channels with varying degrees of interconnectivity upon membrane casting. The water uptake of the Nafion-IPA-H_2_O membrane is higher than that of Nafion-NMP (Table 3). The high degree of agglomeration of macromolecules in the water–alcohol mixture leads to the formation of membranes with a larger pore size distribution, which results in their ability to sorb more water. However, in the case of samples obtained from Aquivion powders, the opposite relationship is observed: water uptake of the Aquivion-NMP sample is higher than Aquivion-IPA-H_2_O. The Aquivion in the IPA-H_2_O mixture has a high degree of agglomeration. It is confirmed by the high viscosity of the dispersion (Table 2). Therefore, when casting membranes, the mobility of macromolecules is limited and the formation of an optimal system of pores and channels is more difficult.

The values of ionic conductivity of the membranes (Table 4) agree with their water uptake. The conductivity of membranes in H^+^ form is 1.3–2.3 times higher than in Na^+^ form. One of the reasons for this is higher water uptake. At the same time, the higher values of conductivity in the H^+^ form are associated with the possibility of proton transfer by the Grottguss mechanism [81]. The ionic conductivity of membranes increases when the functional sulfogroups concentration increase. Therefore, the membranes made from Aquivion PW79S powder dispersion are characterized by maximum conductivity. Their conductivity is 2.5–3 times higher than that of the commercial Aquivion 87 membrane with an SSC. For membranes with a large number of functional groups (EW < 800), the microstructure in the hydrated state changes due to a high degree of swelling. According to [82], if such a membrane is in the hydrated state, its hydrophilic region is a network of connected rods with a diameter of ~1.5 nm. This is probably the reason for the sharp increase in ionic conductivity.

The Donnan exclusion, which characterizes the membrane selectivity to cations, is maintained well enough for all studied membranes in H^+^ and Na^+^ ionic forms. It is proved by the closeness of the slope of the DP dependencies on the negative decimal logarithm of counter ion concentration in solution to the Nernstian slope (Table 4). The Donnan exclusion should increase with increasing concentration of the fixed groups. This pattern is well observed when Na^+^ was a counter ion. For the membranes in Na^+^ form, the slope of the concentration dependencies of the DP increases in the series of Nafion-IPA-H_2_O < Nafion-NMP < Nafion 212 < Aquivion-IPA-H_2_O < Aquivion 87 < Aquivion-NMP (Table 4). The Donnan exclusion increases for the membranes with SSC as compared with the membranes with LSC due to the higher IEC. At the same time, a higher slope is obtained for the Aquivion 87 membrane than for the Aquivion-IPA-H_2_O membrane because of a sufficiently lower water uptake. For the membranes cast from the Nafion dispersions, the slope decreases as compared with the initial Nafion 212 membrane because of the increase in the water uptake and comparable IEC. Higher slope values of the DP concentration dependencies are achieved for the membranes in H^+^ form as compared with Na^+^ form (Table 4), despite their higher hydration. The established slope values (51.2–55.7 mV/p*c*) prove the closeness of the proton transport numbers to 0.9. That is in good agreement with the established high membrane conductivity. In the case of H^+^ form, the slope is comparable for both commercial membranes and Nafion membranes formed by the casting procedure, while it increases for the membranes obtained from the dispersions of Aquivion PW79S powder. It seems that the high concentration of sulfogroups and the formation of a rod-like conducting phase in these membranes [82] leads to an additional exclusion of co-ions from the membrane phase.

### 3.2. Determination of Viral Markers in Artificial Saliva Solutions

**Figure 2:**
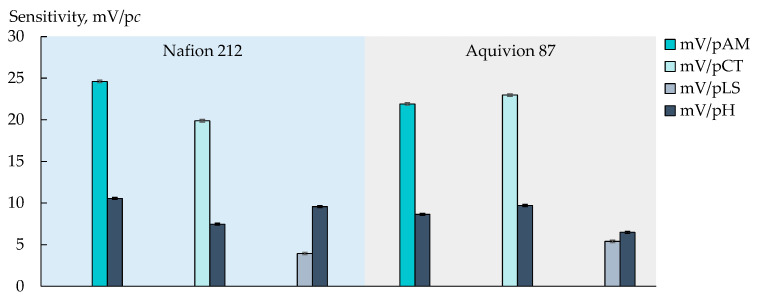
Sensitivity of DP-sensor based on Nafion 212 and Aquivion 87 membranes to ions of viral markers (AM^+/±^, CT^+/±^, or LS^+^) and H_3_O^+^ in artificial saliva solutions.

**Figure 3:**
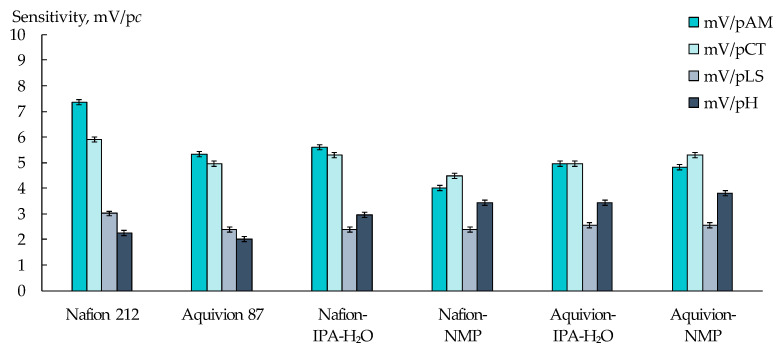
Sensitivity of DP-sensors based on PFSA membranes to ions of AM^+/±^, CT^+/±^, LS^+^, and H_3_O^+^ upon their simultaneous presence in artificial saliva solutions.

The DP-sensor sensitivity to ions of viral markers (AM^+/±^, CT^+/±^, and LS^+^) in artificial saliva solutions in the concentration range from 1.0 × 10^−5^ to 1.0 × 10^−3^ M (Figure 2 and Figure 3) was lower than previously established for the similar analytes in aqueous solutions [72]. This is due to the presence of a relatively high concentration of the supporting electrolytes (1.56 × 10^−3^ M of Na^+^, 5.37 × 10^−5^ M of K^+^, and 1.14 × 10^−5^ M of Ca^2+^). At the same time, the unfavorable influence of pH on the analytical signal decreased owing to the same reason.

The individual influence of each analyte on the DP-sensor response was estimated for commercial membranes (Figure 2). The sensitivity of the DP-sensors to AM^+/±^ and CT^+/±^ was high enough (19.9–24.6 mV/p*c*) and insignificantly differed for both analytes when the Aquivion 87 membrane was used (21.91 ± 0.10 mV/pAM, 22.98 ± 0.10 mV/pCT). At the same time, the sensitivity of the DP-sensor based on the Nafion 212 membrane was higher to AM^+/±^ ions (Figure 2). It can be assumed that this is because the Nafion 212 membrane is characterized by lower water absorption and a smaller pore size. It seems that the transfer of AM^+/±^ ions, which have a branched-chain structure with a hydrophobic moiety, into the membrane pores, limits the availability of sulfogroups for inorganic cations. The latter decreases the influence of the supporting electrolyte on the analytical signal. The DP-sensor sensitivity to LS^+^ ions in artificial saliva solutions was low (Figure 2 and Figure 3). This may be due to the capability of LS^+^ ions to form chelates with Ca^2+^ ions in aqueous solutions [83].

The sensitivity of DP-sensors to ions of viral markers, when they are simultaneously presented in artificial saliva solutions, depended on the type of the initial PFSA polymer and conditions for the membrane formation (Figure 3). The biggest differences in the sensitivity values of DP-sensors to AM^+/±^, CT^+/±^, and LS^+^ ions were achieved using the Nafion 212 membrane. A larger fraction of the main polymer chains in this membrane prevents its swelling, providing various conditions for the sorption of organic ions, which differ in size and hydration level. The increase in the membrane water uptake with the increasing number of functional groups, as well as with the membrane casting, led to the equaling of the DP-sensor sensitivity to AM^+/±^ and CT^+/±^ ions and to an increase in the sensitivity to H_3_O^+^.

**Figure 4:**
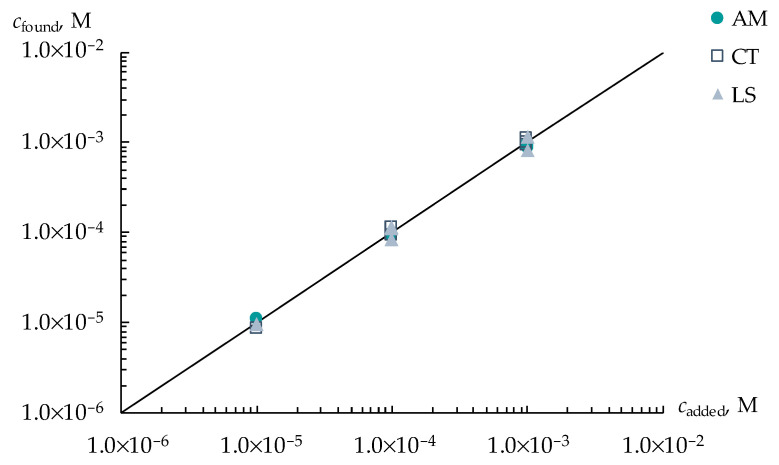
Simultaneous determination of AM^+/±^, CT^+/±^, and LS^+^ ions in artificial saliva solutions with concentrations ranged from 1.0 × 10^−5^ to 1.0 × 10^−3^ M.

A different distribution of the sensitivity of DP-sensors to ions of viral markers and H_3_O^+^ in artificial saliva solutions using various PFSA membranes provided the organization of a multisensory system with low correlations between the responses of single sensors. The array of cross-sensitive DP-sensors based on Nafion 212, Aquivion 87, and Nafion-NMP membranes was tested for the determination of three viral markers in an artificial saliva solution. The use of the multisensory system provided a statistically reliable establishment of the concentrations of AM^+/±^, CT^+/±^, and LS^+^ ions in the range from 1.0 × 10^−5^ to 1.0 × 10^−3^ M (Figure 4). The relative errors of the determination of AM^+/±^ and CT^+/±^ ions were 4–12 and 3–11%, respectively, while the error of the determination of LS^+^ ions was 5–18%. Lower accuracy in the determination of LS^+^ ions was due to the reduced sensitivity of DP-sensors to them because of the interaction with Ca^2+^ ions, which is presented in the composition of the supporting electrolyte. LODs of AM^+/±^, CT^+/±^, and LS^+^ using the developed multisensory system were 1.8 × 10^−6^, 3.0 × 10^−6^, and 0.9 × 10^−6^ M, respectively.

### 3.3. Group Analysis of Viral Markers in Artificial Saliva Solutions

**Figure 5:**
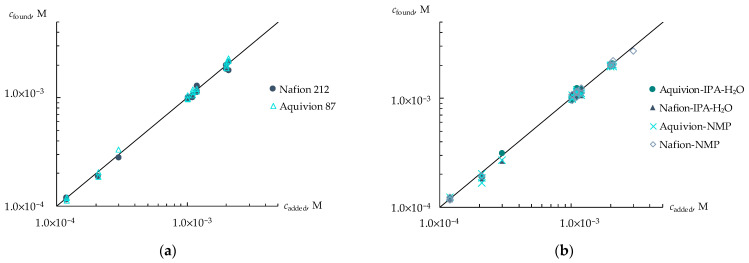
Group analysis of viral markers in artificial saliva solutions using DP-sensors based on commercial membranes (**a**) and membranes obtained by casting procedure (**b**).

The determination of the total concentration of single-type analytes, which are markers of a relevant disorder, is of interest for medical diagnostics. The dependencies of the DP-sensor responses on the overall content of AM^+/±^, CT^+/±^, and LS^+^ ions in artificial saliva solutions had a good predictive ability for all commercial and synthesized membranes (Table 5). They were tested for the group analysis of three viral markers in artificial saliva solutions, in which the concentration of each analyte ranged from 1.0 × 10^−5^ to 1.0 × 10^−3^ M (Figure 5).

The highest accuracy of the group analysis was obtained using Aquivion 87 (the relative error was 0.3–8%) and Nafion-NMP (the relative error was 1.1–9%). This was due to the lowest influence of the pH of the test solution on the DP-sensor response for these membranes.

### 3.4. Regeneration of Membranes and Stability of Calibrations

It is important to control the fouling of the membranes, and the stability of the calibration characteristic of the sensors based on them, while working with organic analytes.

**Figure 6:**
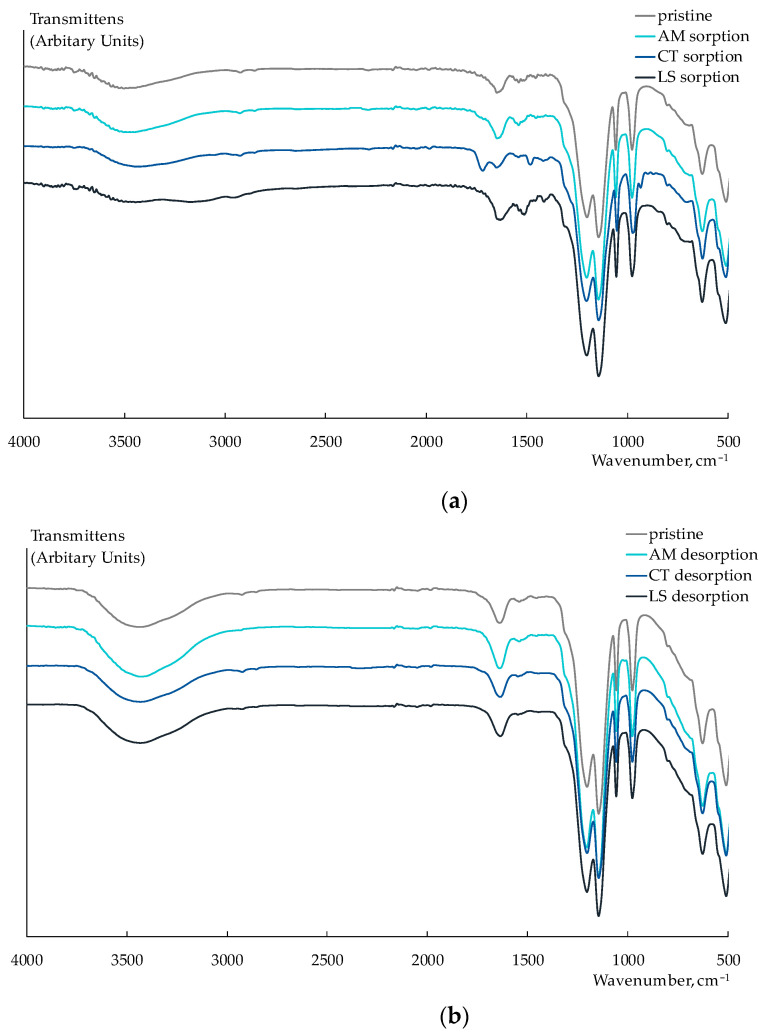
Fragments of FTIR spectra of Nafion 212 membrane after sorption of viral markers from their individual solutions (**a**) and after their desorption (**b**).

To estimate the exposure to fouling, the Nafion 212 membrane was equilibrated with equimolar solutions of NaCl and viral markers (AM, CT, or LS) with the component concentrations of 1 M. These concentrations were two orders of magnitude higher than the concentrations in the test solutions of artificial saliva. After that, the membranes were in-series immersed in 1 M NaCl solution and deionized water for their regeneration. In the FTIR spectra of the Nafion 212 membrane, which was equilibrated with CT or LS solutions, absorbance bands assigned to relevant structural moieties of the analytes can be observed despite the fact that a part of them is overlapped with the intensive absorbance bands of PFSA polymer (Figure 6a).

New bands at 1720 cm^−1^ (C=O stretching vibrations), 1483 cm^−1^ (CH_3_ bending vibrations), 1419 cm^−1^ (the symmetric stretching vibrations of CO_2_^−^), and 935 cm^−1^ (the out-of-plane bending vibrations O–H) appeared in the FTIR spectrum of the Nafion 212 membrane after the sorption of CT^+/±^ ions. A significant broadening and change in the form of peaks in the 3500–3000 cm^−1^ region (N-H stretching vibrations in amino groups) and an appearing band at 1416 cm^−1^ (the symmetric stretching vibrations of CO_2_^−^) were observed in the FTIR spectrum of the Nafion 212 membrane that sorbed LS^+^ ions. In addition, a little shift and some increase in the intensity of the bands at 1632 and 1514 cm^−1^ can be attributed to the asymmetric and symmetric vibrations of N–H, respectively. In the FTIR spectrum of the Nafion 212 membrane equilibrated with the AM solution, bands corresponding to the analyte were not identified. This could be due to the degradation of AM in conditions for the sorption investigation. The FTIR spectra of the membranes after their washing in the NaCl solution are the same as the spectrum of the pristine Nafion 212 membrane. This proves the entirety of the membrane regeneration (Figure 6b).

The lack of fouling of PFSA membranes when working with artificial saliva solutions was also proved by the re-calibration of DP-sensors. The re-calibration, after several months of working, did not reveal any significant changes in the parameters of calibration characteristics (Table 6 and Table 7). The reduced transmembrane transport did not promote the sorption of the analytes into the membrane bulk when performing the measurements with DP-sensors. At the same time, the parts of membranes, which contacted artificial saliva solutions containing viral markers, were effectively regenerated by washing with a NaCl solution.

## 4. Conclusions

The sensory determination of markers of various diseases in saliva is highly promising for the development of non-invasive clinical diagnostics. Membranes based on perfluorosulfonic acid polymers were studied for the development of cross-sensitive DP-sensors for the determination of salivary viral markers (*N*-acetyl-*L*-methionine, *L*-carnitine, and *L*-lysine). Membranes were formed by a casting procedure from dispersions of the polymers with a long (Nafion) and short (Aquivion) side chain in *N*-methyl-2-pyrollidone or water–alcohol mixture. The obtained under laboratory conditions membranes differed from the commercial ones in higher water uptake and ionic conductivity. Moreover, the membranes synthesized from the polymers with a short side chain were characterized by a higher Donnan exclusion due to a larger concentration of functional groups. Thus, the varying of the polymer equivalent weight and the nature of dispersing liquid provided the formation of the materials with sorption and transport properties changed in a wide range. It insured the different distribution of the sensitivity of the DP-sensor based on the commercial and synthesized membranes to *N*-acetyl-*L*-methionine, *L*-carnitine, and *L*-lysine ions in artificial saliva solutions. This made it possible to organize a multisensory system for their simultaneous determination. The simultaneous determination of ions of three viral markers in artificial saliva solution was performed using an array of DP-sensors based on Nafion 212 and Aquivion 87 membranes, as well as membrane cast from the dispersion of Nafion in *N*-methyl-2-pyrollidone. The limits of detection of the analytes in the presence of the supporting electrolyte were (0.9–3.0) × 10^−6^ M. The errors of *L*-lysine determination were lower as compared to other analytes because of its interaction with Ca^2+^ ions, presented in artificial saliva solutions. Moreover, DP-sensors were tested for the group analysis of viral markers. The highest accuracy was obtained for the Aquivion 87 membrane (the relative error was 0.3–8%) and the membrane formed from Nafion dispersion in *N*-methyl-2-pyrrolidone (the relative error was 1.1–9%) due to less pronounced influence of the test solution pH on the analytical signal. The reverse character of the analyte sorption by the membranes was revealed. It provided the effective regeneration of the membranes, and the stability of the calibration characteristics of the sensors based on them, for a long time. Whereas, in most sensors for amino acids and their derivatives, described in the literature, molecularly imprinted polymers or enzymes are used. This fact defines the short lifetime of such sensors. Moreover, the advantage of the proposed methods for the determination of *N*-acetyl-*L*-methionine, *L*-carnitine, and *L*-lysine, which were simultaneously present in artificial saliva solutions, was the lack of the separation and derivatization of the analytes. It is an important difference from most known methods for the determination of amino acids and their derivatives.

## Figures and Tables

**Table 1:** The PFSA polymers used in the work.

Name	Side Chain Composition	Form	EW, g/mol
Nafion 212	-O-CF_2_-CF(CF_3_)-O-CF_2_-CF_2_-SO_3_H	Film	1100
Aquivion E87-05S	-O-CF_2_-CF_2_-SO_3_H	Film	870
Aquivion PW79S	-O-CF_2_-CF_2_-SO_3_H	Powder	790

**Table 2:** Names of samples, conditions for obtaining dispersions, and their viscosity (*η*_disp_., mPa·s at 25 °C).

Name	Polymer	Counter Ion	Dispersing Liquid	Mass Fraction of Polymer, wt%	Dispersing Conditions	*η*_disp._, mPa·s
Nafion-IPA-H_2_O	Nafion 212	Li^+^	IPA-H_2_O 80-20	5.0	80 °C, 2 h	15.1
Nafion-NMP	Nafion 212	Li^+^	NMP	5.0	100 °C, 2 h	11.6
Aquivion-IPA-H_2_O	Aquivion PW79S	H^+^	IPA-H_2_O 80-20	2.5	95 °C, 3 h	37.7
Aquivion-NMP	Aquivion PW79S	H^+^	NMP	2.5	110 °C, 3 h	13.7

**Table 3:** IEC per mass of dry polymer and water uptake of membranes in H^+^ and Na^+^ forms.

Membrane	IEC, mg-eqv/g	Water Uptake, H^+^ Form		Water Uptake, Na^+^ Form	
		W, %	*λ* (H_2_O/−SO_3_^−^)	W, %	*λ* (H_2_O/−SO_3_^−^)
Nafion 212	0.98	29.2	16.5	20.7	11.7
Aquivion 87	1.13	44.0	21.6	32.1	15.8
Nafion-IPA-H_2_O	0.97	42.2	24.2	32.3	18.5
Nafion-NMP	0.97	38.9	22.9	26.8	15.3
Aquivion-IPA-H_2_O	1.23	58.2	26.3	53.5	24.2
Aquivion-NMP	1.23	67.3	30.4	52.5	23.7

**Table 4:** Ionic conductivity (*σ*, mS/cm, the error in estimating was less than 10%) at 25 °C in contact with water and slope (*S*, mV/p*c*, confidence interval was less than 0.6 mV/p*c*) of DP dependence on the negative decimal logarithm of counter ion concentration in solution.

Membrane	H^+^ Form		Na^+^ Form	
	*σ*, mS/cm	*S*, mV/p*c*	*σ*, mS/cm	*S*, mV/p*c*
Nafion 212	4.9	51.4	3.5	50.1
Aquivion 87	6.3	51.6	2.8	52.0
Nafion-IPA-H_2_O	6.8	51.2	5.1	47.5
Nafion-NMP	5.2	52.4	3.4	48.0
Aquivion-IPA-H_2_O	15.6	55.7	7.5	51.1
Aquivion-NMP	17.3	54.9	8.3	52.3

**Table 5:** Calibration characteristics of DP-sensors based on PFSA membranes for the group analysis of viral markers ions in artificial saliva solutions (pY—the negative decimal logarithm of the total concentration of AM^+/±^, CT^+/±^, and LS^+^ ions).

Membrane	*b*_1_, mV/pY	*b*_2_, mV/pH	*ε*, %
Nafion 212	13.07 ± 0.09	5.13 ± 0.06	2
Aquivion 87	10.67 ± 0.08	3.99 ± 0.05	1.6
Nafion-IPA-H_2_O	11.74 ± 0.05	4.83 ± 0.04	1.9
Nafion-NMP	11.05 ± 0.06	3.98 ± 0.04	2
Aquivion-IPA-H_2_O	11.11 ±0.06	4.94 ± 0.04	1.9
Aquivion-NMP	11.48 ± 0.04	5.25 ± 0.03	1.9

**Table 6:** Re-calibration of the multisensory system based on Nafion 212, Aquivion 87, and Nafion-NMP membranes for the simultaneous determination of viral markers ions in artificial saliva solutions.

Membrane	*t*-Test					*t*-Test, *f* = 5, *p* = 0.95	*F*-Test					*F*-Test, *f*_1_ = 2, *f*_2_ = 3, *p* = 0.95
	*b*_0_, mV	*b*_1_, mV/pAM	*b*_2_, mV/pCT	*b*_3_, mV/pLS	*b*_4_, mV/pH		*b*_0_, mV	*b*_1_, mV/pAM	*b*_2_, mV/pCT	*b*_3_, mV/pLS	*b*_4_, mV/pH	
Nafion 212	2.07	0.75	0.72	0.81	0.64	2.57	2.36	1.90	2.37	2.09	2.11	9.95
Aquivion 87	0.48	1.18	0.28	0.92	0.07		1.07	1.13	1.19	0.87	1.18	
Nafion-NMP	1.24	1.39	0.18	0.28	0.52		1.57	1.46	1.72	1.56	1.56	

**Table 7:** Re-calibration of DP-sensors based on Aquivion 87 and Nafion-NMP membranes for the group analysis of viral markers ions in artificial saliva solutions.

Membrane	*t*-Test			*t*-Test, *f* = 5, *p* = 0.95	*F*-Test			*F*-Test, *f*_1_ = 2, *f*_2_ = 3, *p* = 0.95
	*b*_0_, mV	*b*_1_, mV/pY	*b*_2_, mV/pH		*b*_0_, mV	*b*_1_, mV/pY	*b*_2_, mV/pH	
Aquivion 87	0.50	1.46	1.69	2.57	1.53	1.46	1.58	9.95
Nafion-NMP	0.43	0.11	0.55		1.57	1.55	1.56	

## Data Availability

Not applicable.
